# Religion, culture, and discrimination against persons with disabilities in Nigeria

**DOI:** 10.4102/ajod.v5i1.192

**Published:** 2016-10-31

**Authors:** Edwin Etieyibo, Odirin Omiegbe

**Affiliations:** 1 Department of Philosophy, School of Social Sciences, University of the Witwatersrand, South Africa; 2 Department of Educational Psychology, College of Education, Nigeria; 3 Department of Administration and Policy Studies, Delta State University, Nigeria

## Abstract

**Background:**

There is not a lot in the literature on disability in Nigeria concerning the role that religion, culture and beliefs play in sustaining discriminatory practices against persons with disabilities.

**Objectives:**

Many of these practices are exclusionary in nature and unfair. They are either embedded in or sustained by religion, culture and beliefs about disability and persons with disabilities.

**Methods:**

Drawing on various resources and research on disability, this paper looks at these practices in respect of these sustaining factors. Some of the discriminatory practices that constitute the main focus of the paper are the trafficking and killing of people with mental illness, oculocutaneous albinism and angular kyphosis, raping of women with mental illness and the employment of children with disabilities for alms-begging.

**Results:**

The examination of these practices lends some significant weight and substance to the social model of disability, which construes disability in the context of oppression and the failure of social environments and structures to adjust to the needs and aspirations of people with disabilities.

**Conclusion:**

Given the unfairness and wrongness of these practices they ought to be deplored. Moreover, the Nigerian government needs to push through legislation that targets cultural and religious practices which are discriminatory against persons with disabilities as well as undertake effective and appropriate measures aimed at protecting and advancing the interests of persons with disabilities.

## Introduction

In Nigeria, people with disabilities are subjected to numerous types of discrimination (Baba-Ochankpa 2010; Fatunde 2009).^1^ This paper highlights some of these discriminatory practices (some of which constitute human rights abuses) and, in so doing, explores the extent that religion and culture are contributory and supporting factors in the discrimination against persons with disabilities. The practices discussed are the trafficking and killing of people with mental illness, oculocutaneous albinism^2^ and angular kyphosis,^3^ raping of women with mental illness and the employment of children with disabilities for alms-begging.

The conceptual exploration of these practices in the context of religion and culture is important for a number of reasons. Firstly, it motivates for the undertaking of some original empirical research on the relationship between religion and culture and these practices. Secondly, it helps situate the beliefs and attitudes that people in Nigeria hold about disability in the broader context of religion and culture and calls attention not only to the particular beliefs and attitudes but also to the institutions that sustain them. This is particularly important considering a number of scholars have pointed out that an individual’s beliefs about disability constitute foundation blocks upon which that person grounds his or her attitude and behaviour towards people with disabilities (Teaching for Diversity and Social Justice 2007; Madu & Yahaya 2004; Fishbien & Azen 1975; Ozoji 1991; *Thisday News Report* 2010). Thirdly, it shows the sense in which this conceptualisation of the impact of religion and culture on treatment of people with disabilities rests on the social model of disability, according to which disability is understood in the context of oppression and arises due to the lack of societal standards (social environment and structure) in adaptation to the needs and aspirations of people with disabilities (see Hahn 1986:128).^4^

Some of the extant literature with regard to disability and discrimination against persons with disabilities in Nigeria discuss particular forms of disability, focusing on physical facets and when they venture into the moral, social and psychological aspects (Abang 1988; Audu, Idris, Olisah, Sheikh, 2013; Olupona 1991; Omiegbe 2001; Ozoji 1991). Missing from this literature and studies are discussions regarding the relationship between religion and culture and discrimination against persons with disabilities in Nigeria, which is the focus of this paper.

## Notion of discrimination and some discriminatory practices

The term ‘discrimination’ generally refers to separation between things or people based on the recognition and understanding of the difference between one thing and another. In this paper, ‘discrimination’ will be taken to mean the exclusionary practices and the prejudicial treatment of people with disabilities (United Kingdom 1995). Our focus will not only be on basic exclusionary practices against people with disabilities but also on practices that violate the life and personality of people with disabilities. That is, practices that treat them as instruments or *mere things* are what we might call severe human rights abuses.

## Disabilities figures in Nigeria

According to the World Health Organization (WHO 2013), more than a billion people, which is about 15% of the world’s population, have some form of disability.^5^ In Nigeria, the 2006 census put the figure of people with disability at 3 253 169. Of this figure, the total number of women and children with disabilities are 1 544 418 and 1 002 062, respectively. If we go by this figure, then the total number of people with disabilities is approximately 2.32% of the population (140 431 790), with women and children with disabilities being 1.1 and 0.71%, respectively (Federal Republic of Nigeria Official Gazette 2009; National Population Commission 2010). These figures may greatly underestimate the actual number and prevalence of disability in Nigeria. If we take into account the WHO estimate of 15% of any given population having some form of disability or even the point made by Mba (1977) and Abang (1991) regarding the difficulties in achieving a reliable census for those with disabilities in Nigeria, then the total figure of people with disabilities could be around 28 million, given Nigeria’s current estimated population of 188 million.

## Religion, culture and superstition

The term ‘culture‘ has various meanings; however, for the purposes of this paper, it will be taken to mean a set of shared attitudes, beliefs, values, goals and practices that characterises an institution, an organisation or a group (Uwagie-ero, Iseye & Omiegbe 1998). Culture is shared and passed from parents to children or from one generation to another (Eboh & Ukpong 1995). ‘Religion’ can be said to be part of culture and may be defined as a belief in the existence of a deity or a supernatural power, a being that created and controls the universe and who is worshipped on the basis of such belief. ‘Superstition’ can be taken to mean a belief or way of behaving that is based on fear of the unknown or the belief that certain events or things will bring good or bad luck (Merriam-Webster’s Collegiate Dictionary 2003:1255). This understanding of ‘superstition’ takes superstition as an aspect of culture.

## Beliefs and attitudes about disability and persons with disabilities

In the previous section, we identified ‘belief’ as an aspect of culture. ‘Belief’ can be defined as the affirmation or acceptance of a fact, or an opinion accepted as real or true. That is, as ‘the attitude we have, roughly, whenever we take something to be the case or regard it as true’ (Schwitzgebel 2006). A number of beliefs in respect of disability have been isolated in the literature on disability in Nigeria. Abosi and Ozoji (1985), for example, note that beliefs about disability are attributable to different factors such as witchcraft, sex, God, the supernatural and juju.^6^ According to them, these beliefs are generally taken to be the various causes of disabilities. Another aspect of disability beliefs is the one identified by Desta: this is the belief that disability is a curse and people with disabilities are hopeless (1995). Okafor (2003) has also recognised another aspect of disability beliefs. He notes that ‘some local ancient mythology has it that people with disabilities are social outcasts serving retribution for offences of their forefathers’. Munyi (2012), Omiegbe (1998, 2001) and Abang (1988) have also highlighted similar beliefs about disability in other parts of Nigeria as well as in the African context. According to Abang, many people believe that persons with disabilities are not only inferior to those without disabilities but can also be used for social and economic benefits. That is, they lack characteristics that make them full humans and can be used in sacrifices in order to bring wealth or good luck.

## Religious and cultural practices and persons with disabilities

In the following sections, we will discuss some discriminatory practices against persons with disabilities in the context of religion and culture. The practices include the trafficking and killing of persons with mental illness, people with oculocutaneous albinism and angular kyphosis, raping of women with mental illness and the use of children with disabilities for alms-begging.

### Trafficking and killing of persons with mental illness and raping of women with mental illness

People with mental illness are killed as part of rituals, practices that flow from various beliefs that people hold about disability. Many who hold negative beliefs about persons with mental illness claim that their hands are unclean (Omiegbe 1998, 2001). In some communities, it is believed that such persons have committed an abomination, that is, violated the tradition of the communities. In other cases, a mentally ill person is simply labelled as a witch and subsequently burnt to death (Etieyibo 2013; Oko 2003; Omiegbe 2001). This was the case of a middle-aged woman with mental illness in Benin City, Edo State, who was burnt to death by a crowd because of the belief that she was responsible for the various problems facing the community (Houreld 2009; McVeigh 2007; Oko 2003; Purefoy 2010b).

Women with mental illness are also victims of rape in Nigeria. Many are homeless and are often seen on the streets in major cities. According to Dian Blair, the head of Amaudo Itumbauzo, an international non-governmental organisation working with people with mental illness living in poverty in Nigeria, the sexual abuse of women with mental illness ‘is the greatest assault on the rights of female psychiatric patients’ (Eze 2005). In her keynote address at the UN Human Rights Day in Abakaliki, Ebonyi State, Blair noted that there are ritual dimensions to the sexual abuse of many women with mental illness and many of them are raped because of the belief that having intimacy with them could bring wealth or prolong an individual’s life. She further noted that this is unfortunate because the results ‘are the legion of born abandoned children on the streets, who turn out to utterly depend on passers-by for food’.

#### Trafficking and killing of people with oculocutaneous albinism and angular kyphosis

People with oculocutaneous albinism are broadly discriminated in Nigeria. Sometimes they are isolated, and at other times they are trafficked and killed (Oko 2003; Okoro 1975). According to Shehu Shagari, former President of Nigeria, discrimination against people with albinism in Nigeria is endemic and much of the discrimination ‘suffered by people with albinism can be traced to ignorance on the part of the general public’ (El-Kurebe 2010).^7^ Because many people with albinism are targeted for ritual killings, most live in hiding (Anumihe 2008; McVeigh 2007; Nigerian Tribune News Report 2011; Oji 2010; Sky News Report 2008). The killing of people with albinism for rituals is fuelled by the belief that their body parts could be used for portions that will make one wealthy or prolong one’s life (Anumihe 2008; Oji 2010). Two cases came to light recently in South-South Nigeria. In Ugbogui village, a remote farm settlement in Edo State of Nigeria, a person with albinism was beheaded while working in the farm. Similarly, in Abraka Urhuoka Quarters in Abraka community in Delta State, another person was killed while working in his farm. When he was found, some parts of his body were missing (Nigerian Tribune News Report 2011).

People with angular kyphosis are mostly killed for rituals (Omiegbe 2001). There are reports in the local media which suggest that the trafficking of people with this condition is not uncommon.^8^ In 2002, the Nigeria police arrested a man in Ikot-Akpan Abia, Akwa-Ibom State, who traded mostly in parts of people with angular kyphosis and had been in the business for more than a decade. In his confession, he claimed that he sold the parts to herbalists and medicine practitioners for rituals and that kidnapping of people with angular kyphosis is widespread.^9^

#### Religion and culture and the trafficking, killing and raping of persons with disabilities

The discussion on the trafficking and killing of people with mental illness, oculocutaneous albinism and angular kyphosis, and raping of women with mental illness highlights that these practices are done as part of rituals. According to reports in some Nigerian newspapers, a number of missing persons (many of which include persons with disabilities) in various cities and communities in Nigeria are kidnapped, trafficked and killed for rituals (Next.com News Report 2009). In one report presented by Odejobi (2010), individuals who were fortunate to escape from their kidnappers recounted stories of how people that were kidnapped were killed for ritual purposes. Such ritual killings have either a personal or a communal dimension [i.e. done in order to cleanse the community from some sin or evil claimed to have been committed by people with disabilities or other community members (Nigerian Tribune News Report 2011)]. Given this common knowledge, persons with disabilities for the most part live, eat and sleep in fear (Odejobi 2010). In support of these reports about the killing of people with disabilities, Emmanuel Ojukwu, the public relations officer for the Nigeria police force, in an interview with the *News Agency of Nigeria* made the point that many kidnapping cases in Nigeria result in the dismemberment of bodies for rituals (Next.com News Report 2009).

### Employing children with disabilities in alms-begging

In Nigeria as in many other parts of Africa, parental authority is respected and highly esteemed. A child that resents his or her parents or parental authority in general is not only criticised but also severely punished. Such punishment sometimes includes being spanked, deprived of some necessities, locked up in a room and grounded for days or weeks. Because of the authority that parents have over children and the cultural value placed on respect for such authority, it is easy for parents of children with disabilities to send them out onto the streets to beg for alms (Omiegbe 1995). Part of the appeal in using children with disabilities in this way is the thought that the sight of such children is quite likely to evoke a sense of sympathy from members of the society, especially from those that take alms-giving as an obligation.

Parents or guardians of children with disabilities, who send their children out for alms-begging, compare what they do with parents or guardians that send out their children as street traders. Children with disabilities who refuse to beg are usually threatened with beating or refused food. There are cases where the punishment for refusal is more severe, for example, chasing the child with disabilities away from home. In order not to suffer this fate, children usually accede to the wish of their parents. By and large, it could be surmised that children with disabilities obey their parents because of fear of being punished (Omiegbe 1995).

#### Religion and culture and employing children with disabilities in alms-begging

Many parents who send their children with disabilities to beg for alms do so for economic reasons. Some of these ideas have been explored by Omiegbe (1995). There are also cultural and religious aspects to the practice of using children with disabilities for alms-begging. Dunapo (2002) notes that alms-begging in general has religious and cultural dimensions. He further states: ‘Begging is also a human problem involving not only the disabled persons but also refugees from war ravaged countries. It has *religious and cultural connotations* [*emphasis added*].’

The religious and cultural aspects to alms-begging and the use of children (with or without) disabilities to beg for alms can be seen from the age-old Almajiri system, which is an ancient tradition and cultural practice. The Almajiri is an aspect of the Northern Nigerian Islamic Education System, that is, a ‘semi-formal non-secular education in which children between the ages of 4 and 18 are assigned to wandering Islamic teachers usually referred to as Malams’ to learn the Koran and also to acquire some form of Islamic knowledge (Yusha’u, Tsafe, Babangida & Lawal, 2013:127). Once in the schools, and often hundreds of miles away from their families, they receive little education and money and, thus, generally have to beg to survive. As Purefoy (2010a) notes, these children are frequently seen flooding the streets with small bowls; ‘across the north, an afternoon break in classes sends the children flooding into the streets with small bowls to search for scraps’.

Yakasi and Amupitan (particularly Amupitan) have discussed how religion and culture in the area of the Almajiri system is related to alms-begging. Yakasai (1990), for example, notes that the Islamic religion encourages the practice of alms-begging and some of the attempts at justifying the practice are unconvincing. Like Yakasi, Amupitan (2001) claims that the Almajiri system promotes begging. He argues that the reason begging is lucrative in Damaturu, the capital of Yobe State (Northern Nigeria), is that it has the Almajiri culture which encourages begging. He notes:

The Almajiri system is very widespread in Yobe State as in most States of the north which is believed to be a product of the Koranic system into which some parents dump their male children and forget them there. The poor little brats are abandoned to the care of the Koranic teacher who is not earning any salary. The poor teacher simply engages the pupils in part-time begging, the proceeds from which is used to pay the teacher and feed the pupils. In most cases the Almajiri (the Koranic pupil) does not know his parents, his home or any of his relations. He is entirely on his own and ends up on graduation a beggar.

One may conclude that the Islamic practice of Almajiri sanctions begging, as do some passages in the Koran.^10^ Accordingly, it is no wonder that it is common to see children with disabilities in many cities in Nigeria being used by their parents or guardians in alms-begging. Given the Koranic/Islamic basis of alms-giving and alms-begging, adult persons with disabilities who are Muslims and who see alms-begging as some ‘religious duty’ do not waver going into the streets themselves to beg or send their children with disabilities onto the streets to solicit for alms. Simply put, the Islamic religion and the Almajiri culture sustain the practice of using children with disabilities to beg for alms – a practice that is discriminatory against children with disabilities. This claim can be teased out in two ways. Firstly, the Islamic religion and the Almajiri culture sustain the practice of using children with disabilities to beg for alms in the context of the Islamic injunction and the Almajiri system where alms-begging is justified and legitimised. Secondly, the use of children with disabilities for alms-begging invokes certain psychological responses in people. That is, people who are moved by the condition of children with disabilities feel a compulsion to help by giving them material and financial resources. These benefits that come to both children with disabilities and their parents come about only because alms-begging and the practice of using children with disabilities to beg for alms are sustained by the Islamic religion and the Almajiri culture.

If the above reports and evidence are accurate, then they highlight the point about the role of culture in discriminatory practices against persons with disabilities, which seems consistent with Oliver’s variant of the social model of disability. In his seminal work, Oliver seeks to provide conclusive evidence that disability ‘as a category can only be understood within a framework which suggests that it is culturally produced and socially structured’ (Oliver 1990:22). The point is that the discriminatory practices against persons with disabilities are sustained by culture and in doing so reinforce the dominant perspectives regarding disability. Therefore, it is not only the case that people with disabilities are killed on the basis of disability beliefs (e.g. superstition), but these killings are also ritualised; they are ritualised because they arise from a particular culture embedded in a particular worldview. The killings of people with disabilities in Nigeria can be considered aspects of cultural practices or culture. Perpetrators pick out such persons and kill them because they either believe that doing so would make them rich and successful or believe it as part of some ceremony rituals. This is the point that we made above about the communal dimension of ritual killings and is in line with Abang’s remark that people with disabilities are targeted for their social and economic benefits, that is, killed for their utility value (1988).

With regard to religion, Olupona (1991) has noted that various ritual practices carried out in many communities across Nigeria are grounded in some form of African traditional religion (ATR). This claim is important considering that disability beliefs and the attitudes towards people with disabilities may not explicitly be sanctioned by Christianity and Islam – the two dominant religions in Nigeria. But then one may ask if many Nigerians are professed Christians and Muslims, how come they have some deep connection and attachment to practices and activities that are wedded to ATR? Oyebode’s (2009) answer to this question is that it has to do with the strong influence exerted by ATR, and this influence is seen in the attitudes of Nigerians towards oaths administered via the Holy Bible and the Holy Koran and the ATR. The point to be taken from Olupona and Oyebode is that even though western education is a socialising agency, as Amposah (1975) has noted, the fact that the ATR still holds some attraction among many may have to do with Dzobo’s (1974) point that the individual’s being seems connected to him identifying with the fortunes of his group and [feeling] deeply rooted in the corporate being of his society. The point is that many Nigerians that are still wedded to the ATR are goaded by beliefs embedded in this religion to engage in ritual killings of persons with disabilities.

One can conclude that both religion and culture exert powerful influence on many Nigerians not only when it comes to negative attitudes towards people with disabilities but also in their engagement in highly discriminatory practices against them.

## Conclusion

Our discussion has highlighted that religion and culture promote certain beliefs and attitudes about disability and people with disabilities that lead to discriminatory practices. That is, they are sustaining factors in discrimination against people with disabilities. These practices should be wholeheartedly denounced because they result in the invasion of the dignity, personhood and life of persons with disabilities. They trespass the inalienability of human rights, in general, and the rights of persons with disabilities, in particular, as recognised by human rights conventions and laws (United Nations, Convention on the Rights of Persons with Disabilities). To ensure that persons with disabilities are treated fairly and to combat stereotypes, prejudices and harmful practices relating to persons with disabilities, the Nigerian government would have to push through legislation that can target cultural and religious practices which are discriminatory against persons with disabilities. In addition, it has to undertake effective and appropriate measures aimed at raising awareness throughout the society about persons with disabilities.

## Footnotes

1 Historical examples of discrimination against people with all forms of physical and mental disabilities range from being ridiculed to being condemned to permanent exclusion in asylums, from being allowed to drown or die to being executed (Pritchard 1963:15–30: See also Teaching for Diversity and Social Justice 2007). As Barton has noted, throughout antiquity people with disabilities have been subjected to a multiplicity of oppressive social attitudes, which have included ‘horror, fear, anxiety, hostility, distrust, pity, over-protection and patronizing behavior’ (Barton 1993:8). According to Barnes (1991), the real concern of disability, therefore, has to do with these pejorative attitudes combined with particular social contexts, namely, an inhospitable physical environment (e.g. inaccessible buildings and unusable transport systems).

2 Oculocutaneous albinism is a group of inherited disorders of melanin biosynthesis or a congenital disorder which is characterised by a generalised reduction in pigmentation (called melanin) of the skin, hair and eyes (Christianson, Howson & Modell 2006; Grønskov, Ek & Brondum-Nielsen 2007; Okoro 1975; Winship 2003).

3 Angular kyphosis is a common condition of a curvature of the upper spine and can be the result of either degenerative disease (such as arthritis) or developmental problems (Omiegbe 2001).

4 See also Charlton (1998:27) and Abberley (1987:163).

5 For the classification of disability and health see World Health Organization (2011).

6 Juju refers to charms, amulets and spells used in some parts of West Africa as a part of witchcraft or to affect others or events, negatively or positively.

7 Shagari made this remark during the 4th National Conference on Albinism in Sokoto State on 12 July 2010.

8 Some relatively recent examples of trafficking of people with angular kyphosis include the following: (1) the killing of a famous herbalist, a male angular kyphosis sufferer, in Benin City, Edo State, Nigeria (Omiegbe 2009); (2) the killing of a 22-year-old female angular kyphosis sufferer, Taibat Oseni, in Osun State, which implicated a Nigerian senator. According to the Osun State Police Command, Oseni was kidnapped from her home and then taken to a 15-year-old abandoned building owned by a senator where she was killed and her protrusion removed (Dike 2009; Kolawole 2009).

9 According to him, a person with angular kyphosis attracts the sum of N400 000 (US$2100) (Nkanga 2002).

10 See Koran 2:273 and Koran 42:27.
